# Capecitabine-loaded NLC for Breast Cancer Treatment: Preparation, Characterization, and *In vitro* Evaluation

**DOI:** 10.2174/0115672018309370240708113038

**Published:** 2024-07-29

**Authors:** Muhammad Hadi Sultan, Yosif Almoshari, Syam Mohan, Mohamed Ahmed Al-Kasim, Hamad S. Alyami, Mohammad Azam Ansari, Mohammad Intakhab Alam

**Affiliations:** 1 Department of Pharmaceutics, College of Pharmacy, Jazan University, Jazan, Saudi Arabia;; 2 Substance Abuse and Toxicology Research Centre, Jazan University, Jazan, Saudi Arabia;; 3 Department of Pharmacology, College of Pharmacy, Jazan University, Jazan, Saudi Arabia;; 4 Department of Pharmaceutics, College of Pharmacy, Najran University, Najran, Saudi Arabia;; 5 Department of Epidemic Disease Research, Institute for Research and Medical Consultations (IRMC), Imam Abdulrahman Bin Faisal University, Dammam, Saudi Arabia

**Keywords:** Capecitabine, NLC, stability studies, tween 80, 5-fluorouracil, sodium dodecyl sulphate

## Abstract

**Background:**

Cancer treatment often involves the use of potent antineoplastic drugs like Capecitabine (CAP), which can lead to serious toxicities. There is a need for dosage forms to manage these toxicities that can deliver the medication effectively to the target site while maintaining therapeutic efficacy at lower doses. To achieve the aforesaid objective, NLC containing capecitabine (NANOBIN) was prepared and evaluated. Different formulations of NANOBIN, denoted as CaTS, CaT1S, CaT2S, CaTS1, and CaTS2, were designed and evaluated to improve drug delivery and therapeutic outcomes.

**Methods:**

The NANOBIN formulations were prepared using the hot homogenization method. The characterization of these formulations was conducted based on various parameters such as particle size, Polydispersity Index (PDI), Zeta Potential (ZP), Transmission Electron Microscopy (TEM) imaging, and Encapsulation Efficiency (EE). *In vitro* evaluations included stability testing, release studies to assess drug release kinetics, and a cytotoxicity assay (MTT assay) to evaluate the efficacy of these formulations against human breast cancer cells (MCF-7).

**Results:**

The characterization results revealed that all NANOBIN formulations exhibited particle sizes ranging from 65 to 193 nm, PDI values within the range of 0.26-0.37, ZP values between 46.47 to 61.87 mV (-ve), and high EE percentages ranging from 94.121% to 96.64%. Furthermore, all NANOBIN formulations demonstrated sustained and slow-release profiles of CAP. The MTT assay showed that the NANOBINs exhibited significantly enhanced cytotoxic efficacy, approximately 10 times greater than free CAP when tested on MCF-7 cells. These findings indicate the potential of NANOBINs to deliver CAP effectively to the target site, enabling prolonged drug availability and enhanced therapeutic effects at lower doses.

**Conclusion:**

The study demonstrates that NANOBINs can effectively deliver CAP to target sites, prolonging drug exposure and enhancing therapeutic efficacy while reducing the required dose. Further studies are necessary to validate these findings and establish NANOBINs as a preferred treatment option for cancer therapy.

## 1 INTRODUCTION

The standard approach to treating cancer is hampered by the drug's short half-life and low bioavailability, as well as the need for a high dose, difficulty in delivering enough to the tumor site, and increased toxicity and adverse effects [1, 2]. Novel drug carriers, such as lipid nanocarriers, might be a preferable option to achieve greater therapeutic efficacy. Together with cancer, lipid nanocarriers (including NLC), as well as other nanocarriers, have shown enormous potential for efficient drug delivery in a wide range of diseases. The appropriateness is based on the benefits of using lipid nanocarriers above conventional therapy for the treatment of cancer (such as resistance to chemotherapy and the associated adverse effects). Moreover, the advantages associated with the specific properties of lipid nano-carriers encompass enhanced internalization and intracellular transportation of drugs, targeted deposition of drugs in tumors, improved pharmacokinetics and pharmacodynamics, and mitigated adverse effects through the reduction of drug bio-distribution [3-5]. Lipid nano-carriers, along with nanostructured lipid carriers (NLCs), have been widely employed as delivery systems for various therapeutic substances in previous studies. These substances include all-trans retinoic acid [6], epigallocatechin gallate [7], citral [8], gefitinib [9], and imatinib [3]. NLCs consist of both solid lipids, namely fats, and liquid lipids, namely oils [10]. Nanocarriers based on Nanostructured Lipid Carriers (NLCs) have superior loading capacity and durability in comparison to other lipid nanocarriers, such as Solid Lipid Nanoparticles (SLNs), which only consist of solid lipids [11-13].

Capecitabine (CAP, Fig. **S1**) is a white to off-white-colored powder with a molecular weight of 359.3 g/mol. The drug is very soluble in methanol, ethanol, acetonitrile, and water (26 mg/mL, FDA). It is an orally administered prodrug intended for the treatment of colorectal cancer, breast cancer, and gastric cancer [14, 15]. It acts by inhibiting DNA synthesis and slowing the growth of tumor tissue after enzymatic conversion to 5-fluorouracil (5-FU). The de novo synthesis of DNA needs thymidine phosphate, which 5-FU inhibits. It belongs to the fluoropyrimidine class of medications, which are the backbone of the chemotherapeutic agents used for the treatment of various types of cancer [16-18]. Annually, these types of drugs are received by more than 2 million patients [19, 20]. The incidence of severe to life-threatening toxicities was reported in 10 to 40% of patients [19]. The common dose-limiting adverse effects associated with CAP include myelosuppression, hyperbilirubinemia, stomatitis, dermatitis, diarrhea, fatigue, and nausea [21]. It is readily absorbed from the GIT (gastrointestinal tract), and its recommended daily dose is 500 mg with a short elimination half-life of not more than 1 h [19, 22]. The dose-related toxicity of anticancer drugs (including CAP) needs to be managed for better therapeutic efficacy. Moreover, the twice-daily administration of CAP (Xeloda^®^) leaves a gap of approximately 6 hours. This much of a gap is undesirable clinically for antitumor treatment [23-25]. So, the complications opened the door for the development and evaluation of other dosage forms that can provide CAP for a longer duration at the site of action at a reduced dose. In view of that, targeted delivery using extended-release oral dosage forms [23], PLGA nanoparticles [26], and nanopolymeric micelles [27] were studied and evaluated. In furtherance, NLC can be a dosage of choice with better stability and biocompatibility, in addition to providing CAP for a longer duration at the site of action at a reduced dose.

The current study's objective was to develop and assess NLCs that contained CAP(NANOBINs). These were prepared using the hot homogenization procedure, and they were tested for stability and release studies and characterized for particle size, zeta potential, and polydispersity index. At 25°C (room temperature), the stability investigations were carried out for a period of three months. To assess the antineoplastic potential of NANOBINs, the cytotoxicity assay was carried out.

## 2 MATERIALS AND METHODS

Capecitabine was bought from Dr. Reddy's Laboratories Ltd. (Unit VI, AP, India). Stearic acid (SA) was purchased from Himedia in Mumbai, India. Tween-80 (T80) and sodium dodecyl sulfate (SDS) were purchased from Loba Chemei (Mumbai, India) and PanReac AppliChem (Darmstadt, Germany), respectively. A local shopkeeper in the Saudi Arabian province of Jazan provided the sesame oil. All other substances, including reagents, were of analytical grade.

### 2.1 Preparation

The NLC that contains CAP (NANOBIN) was made by homogenizing the lipid phase with the aqueous phase that contains surfactants while the temperature was elevated. In a beaker, 25 mg of capecitabine (CAP) was dissolved in a combination of molten stearic acid (SA, 500 mg) and 200 ❍L of sesame oil (SO). This was carried out to produce the lipid phase. To prepare the aqueous phase, 25 mL of water containing tween-80 (T80) and sodium dodecyl sulfate (SDS) in ratios that varied according to weight were added (Table **1**). Both phases were brought to a temperature of 70°C. The aqueous phase was then transferred to the beaker that contained the lipid phase, and using a homogenizer (HG-15D, WiseTis, Germany), it was homogenized for 20 minutes at a speed of 6000 rpm. After the mixture had been homogenized, it was filtered using Whatman filter paper (Sigma-Aldrich), and was kept so that it could be utilized in the following investigations.

### 2.2 Characterization and Evaluation

#### 2.2.1 Particle Size, Zeta Potential (ZP), and Polydispersity Index (PDI)

A zetasizer (Malvern, Nano ZS90, UK) was utilized to carry out the size analysis and PDI on the NANOBIN. Before performing the analysis, the NANOBIN formulations were suitably diluted with water to eliminate the possibility of multi-scattering events. With the use of the same zeta sizer, the Zeta Potential (ZP) of the surface charge of NANOBIN was determined. The results of each measurement were recorded three times.

#### 2.2.2 Encapsulation Efficiency

The ultrafiltration-centrifugation technique was utilized to calculate the Entrapment Efficiency (EE) for NANOBIN formulations. The Amicon Ultra-2 mL, 3K ultra-filter tube, manufactured by Millipore in Ireland, was utilized in this experiment. It was loaded with 1 mL of the NANOBIN formulation, which contained about 2 mg of CAP. Subsequently, centrifugation was conducted at a speed of 4000 × g (6861 rpm) for 20 minutes at a temperature of 25°C. The resulting filtrate was further diluted with Millipore water and thereafter subjected to spectrophotometric analysis. The quantification of unentrapped CAP in the filtrate was conducted by assessing the absorbance at a wavelength of 241 nm with a UV-visible spectrophotometer (Shimadzu, Kyoto, Japan). The percentage encapsulation efficiency (% EE) of CAP within NLC was determined using the following formula:

% EE = 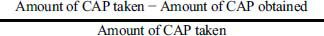 X100

#### 2.2.3 Surface Morphology

High-resolution techniques such as Transmission Electron Microscopy (TEM) and Scanning Electron Microscopy (SEM) were employed for the analysis of the morphology and structural characteristics of nano-formulations. The prepared formulation was subjected to characterization using a transmission electron microscope (Morgagni 268, FEI, Czech Republic) with an acceleration voltage of 80 kilo electron volts (keV). To get visual depictions, a small quantity of the dispersed solution containing the prepared formulation was put over TEM grids (400 mesh) that had been coated with a thin coating of carbon film. The TEM grids underwent a drying procedure before being positioned on the microscope for subsequent examination.

#### 2.2.4 Freeze Drying

Mannitol solution (5 g dissolved in 100 mL of water) was used as a cryoprotectant to avoid any damage caused by the freezing effect. NANOBIN formulations were mixed with mannitol solution (1:4) and transferred to a 250-mL glass jar. The freezing process involved placing the sample in an ultra-low-temperature freezer (U9280-0002, New Brunswick Scientific, Edison, NJ, USA) and maintaining it at a temperature of -70 °C for a duration of 12 hours. The frozen sample was affixed to a laboratory freeze dryer (model BT85, Millrock Technology, NY, USA) in order to undergo the process of lyophilization. The dried samples were gathered after a 24-hour drying period and subsequently kept for further utilization.

#### 2.2.5 DSC Analysis

The Differential Scanning Calorimetry (DSC) method was utilized to do thermal analysis. Using Q200 DSC equipment (made in the USA by TA Instruments), freeze-dried NANOBIN samples were analyzed to identify the temperatures at which the melting point occurs. A sample of NANOBIN weighing approximately 2 mg was placed in an aluminum pan, was then heated to 250°C at a rate of 10 °C per minute while being purged with nitrogen gas. An empty aluminum pan was utilized as a point of reference for all the measurements. The TRIOS management program was used to analyze the graphs that were produced. The values for the melting point were found by finding the point where the relative tangents intersected with the baseline. The crystallization index (% CI) was calculated as per the formula given below to determine the crystalline state of the drug in the formulation [28].

CI [%]= 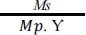


Where Ms is the melting enthalpy (J g^–1^) of NLC, Mp is the melting enthalpy (J g^–1^) of pure solid lipid, and Ƴ represents solid lipid concentration (%) in NLC dispersion.

#### 2.2.6 In vitro Release Study

For the *in vitro* release test, the dialysis bag technique was used. After the end of the dialysis bag (12-14 KD MWCO, Spectrum Laboratories, CA, USA) was tied off, 2 mL of the NANOBIN formulation was added, which is equivalent to 2 mg of CAP. After transferring the formulation, the bag was knotted at the other end. The filled bag was then placed in a beaker containing phosphate buffer (pH 7.4) as release media. The release medium was kept at 37 °C and agitated at 50 revolutions per minute with a magnetic bead. A quantity of 2 mL was extracted from the beaker at time intervals of 0.5, 1, 2, 4, 6, 12, and 24 hours and afterwards transferred into pre-labeled test tubes. The volume of the release media was consistently maintained by promptly replacing it with an equal volume of new buffer. The samples that were gathered underwent filtration using a syringe equipped with a 0.45 µm filter. The filtered samples were then subjected to analysis for drug content using a UV-visible spectrophotometer (Schimadzu, Kyoto, Japan). The analysis was conducted at a wavelength of maximum absorption (λ_max_) of 241 nm.

#### 2.2.7 Stability Study

The stability of NANOBINs was evaluated after storing them for 3 months at room temperature (25°C). Each NANOBIN formulation was then stored in different glass containers. All stored NANOBINs were examined and assessed periodically for physical changes, including agglomeration, color, consistency, and smell. Furthermore, the stability was evaluated in terms of characterization parameters that included particle size, PDI, EE, and ZP.

#### 2.2.8 Cell Culture

In this investigation, metastatic human breast cancer MCF-7 cells were procured from ATCC. The cells that were acquired were cultured in RPMI 1640 medium (Gibco, Grand Island, NY, USA), which was supplemented with 10% fetal bovine serum (Gibco, Paisley, UK) and 1% streptomycin and penicillin antibiotics (Sigma Aldrich, St. Louis, MO, USA). The cells were incubated at a temperature of 37°C in a carbon dioxide (CO_2_) incubator (New Brunswick, Scotland) while maintaining a pH of 7.4.

#### 2.2.9 Cell Viability Assay (MTT)

To estimate the cell death-inducing capacity of various formulations, an MTT assay has been performed [29, 30]. In a concise manner, cells were seeded into 96-well culture plates at a density of 10,000 cells per milliliter. The cells were cultured and incubated in a CO_2_ incubator for 24 hours to facilitate growth and attachment. On the second day of the experiment, the plates were removed from the incubator, and the culture medium was substituted with new media and formulations. Different concentrations of formulations were introduced to the cells, with the greatest dosage being 200 µg/mL. The plates that had been treated with the formulation were incubated for another 72 hours. Following the completion of the treatment, a volume of 20µL of MTT dye (5 mg/mL, Invitrogen Corporation, San Diego, CA, USA) was introduced into each well and subsequently incubated for an additional duration of 4 hours. Upon completion of the incubation period, the media were meticulously extracted from the plates, ensuring that the formazan crystals produced at the plate's base remained undisturbed. Subsequently, 100 µL of dimethyl sulfoxide (DMSO, Fischer Chemicals, UK) was introduced into each well to facilitate the dissolution of the crystals. The final product, exhibiting a violet hue, was further analyzed at a wavelength of 570 nm using a microplate reader (BMG LABTECH, SpectroStar Nano, Ortenberg, Germany). The percent vitality of the cells was determined by calculating it from the appropriate control values using the method provided below based on the raw data. The trials were conducted in triplicate.

Growth inhibitio*n* = 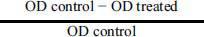 X 100

### 2.3 Statistical Analysis

The results in the current investigation werereported as the mean value accompanied by the standard deviation (SD). A one-way analysis of variance (ANOVA) was employed to compare the means of various treatment groups. A significance level of *p <* .05 was deemed to be statistically significant.

## 3 RESULTS

### 3.1 Particle Size, Polydispersity Index (PDI) and Zeta Potential (ZP)

NANOBINs were successfully prepared and evaluated. Frequency graphs of size and ZP are shown in Fig. (**1**). Table **2** displays the outcomes of the characterization parameters. The size of all NANOBINs was determined to be less than 200 d.nm. The largest average size was found to be 120.31 d.nm for CaTS2, and the smallest one was 65.5 d.nm for CaT2S.

The PDI of all NANOBINs is shown in Table **2**. The highest value of PDI was observed for CaTS1, followed by CaTS, CaT2S, CaT1S, and CaTS2 (*i.e*., CaTS1˃CaTS˃ CaT2S˃CaT1S˃ CaTS2). The PDI of all NANOBINs was found to be less than 0.5.

Zeta Potential (ZP) of all NANOBINs is shown in Table **2**. The ZP value was found to be negative. All NANOBINs exhibited a high value of ZP (˃ 20). CaT1S exhibited the highest value of ZP followed by CaTS2, CaTS1, CaT2S, and CaTS (*i.e*., CaT1S˃CaTS2˃CaTS1˃CaT2S˃ CaTS).

### 3.2 Encapsulation Efficiency (EE)

The amount of free CAP and CAP incorporated into the lipids of NLC was determined by the EE of NANOBINs. The EE of all NANOBINs was found to be greater than 90% (Table **2**). The high value of EE indicates the lipophilic nature of CAP. Moreover, the high value of EE ensures that a high amount of CAP is transported into the target cells.

### 3.3 Surface Morphology

All NANOBINs were employed to study morphology by taking images with the help of TEM and SEM. The images are shown in Fig. (**2**). The images were observed to be uniform in size and spherical in shape. The NANOBINs in the images appear to be in the range of nanometers. Some images exhibited aggregation, which may be due to the short drying time given to the samples before taking the images.

### 3.4 DSC Analysis

DSC results were obtained for CAP (drug), stearic acid (fat), mannitol (cryoprotectant), and NANOBINs (CaTS, CaT1S, CaT2S, CaTS1, CaTS2, Fig. **S2**). The melt of an amorphous state (a less ordered crystal) requires less energy or no energy than the melt of a perfect crystalline substance. It is attributed to the smaller amount of energy needed to overcome the lattice force.

The decrease in enthalpy of the lipid matrix with respect to the pure materials is measured as an indication of its amorphous state. The enthalpy values of different materials are shown in Table **3**. The enthalpy of NLC formulations was found to be lower (< 50 J/g) than that of pure lipid (stearic acid, 222.5 J/g). Thus ensuring the amorphous state of the lipid matrix in NLC formulations (*i.e*., NANOBINs). All NANOBINs exhibited the absence of a CAP endotherm peak, confirming complete solubilization in lipids and entrapment. It is supported by the results of EE (> 90%) and release studies (controlled release). The CI of NANOBINS was determined to be 22.45, 14.18, 11.88, 4.47, and 17.71 percent for CaTS, CaT1S, CaT2S, CaTS1, and CaTS2, respectively.

### 3.5 *In vitro* Release Study

All NANOBINs underwent *in vitro* release tests utilizing the dialysis bag method. The pattern of release shown by NANOBINs is displayed in Fig. (**3**). The released amount of CAP in the release media was estimated at different time intervals over the course of 24 hours. The release media was maintained in sink condition throughout the study by selecting and maintaining the appropriate volume. The intervention due to CAP solubility can be avoided by maintaining sink conditions. The release pattern of NANOBINs did not demonstrate any burst effect. The occurrence of the burst effect is commonly attributed to the existence of non-encapsulated drugs on the surfaces of nanostructured lipid carriers (NLCs), [31]. Here, the absence of the burst effect specifies the absence of unentrapped drugs. As a result, the bulk of CAP is judged to be present within the core of NLC particles. Conveniently, it was supported by high EE values (> 90%) of NANOBINs. Furthermore, since the majority of CAP was occupied within the core of NLC, it took longer to transport the CAP out of the NLC particles. Therefore, it encouraged NANOBINs to exhibit slow and sustained release behavior. The prolonged-release characteristic of NANOBINs may also be attributed to several other factors. Partitioning between the lipid matrix and water and the uniform trapping of CAP across the system may be responsible for this. Furthermore, the solid matrix of NLC and subsequent immobilization, plus the barrier function of the interfacial membrane, strengthen the sustained release behavior of NANOBINs [9, 32, 33]. As a result, NANOBINs support the need for a single dose to be sufficient for a longer duration of antineoplastic effect in cancer treatment. It will provide less frequent administration, less exposure, improved compliance, and be more patient-friendly.

The release mechanism of CAP from NANOBINs was analyzed and is displayed in Fig. (**4**). Different release models, including zero-order, first-order, Higuchi, and KP models, were used to explain the release mechanism. The value of squared R (*R^2^*) was determined for each model and used to explain the mechanism for the release of CAP from NANOBINs. The highest R^2^ value was considered for the model, followed by NANOBINs. Multiple release mechanisms were exhibited by the NANOBINs. CaTS, CaT1S, and CaTS2, were all found to reveal zero-order, first-order, and KP models for the release CAP. However, the zero-order release mechanism was followed only by CaT1S (*R^2^* = 0.975) and CaTS2 (*R^2^* = 0.980).

### 3.6 Stability Study

Stability assessments were conducted on all NANOBINs for three months under ambient conditions at a temperature of 25 °C. Results are shown in Table **4**. In addition to particle size and PDI, ZP was also found to be affected by storage. An increase in particle size was seen in the instances involving CaTS, CaT1S, and CaT2S when analyzed after storing them for a three months. The increase in size is affected by the amount of T80 used in the preparation of CaTS, CaT1S, and CaT2S. A higher amount of T80 in CaT2S (100 µL) resulted in an increase in the growth in size (size difference = 35.47) as compared to the NANOBINs containing lesser amounts of T80 in CaT1S (difference = 33) and CaTS (difference = 26) after 3 months. Minimum growth in size was observed in CaTS containing the lowest amount of T80 (50 µL, *i.e*., CaT2S˃CaT1S˃CaTS, *p* ˂ .05).

Among other parameters studied in the present study, the appropriateness of nano-formulations for a certain drug delivery route is also determined by the PDI. The storage period visibly affected the PDI of NANOBINs (Table **5**). The PDI was found to decrease for all NANOBINs after 2 months of storage, except for CaT1S. However, an increase in the PDI values was observed after 3 months of storage, except for CaT1S. The disparity in PDI of NANOBINs may be attributed to the variation in particle size after 3 months of storage. The minimum value of PDI was obtained for CaT1S, and the maximum value for CaTS after storage. Thus, based on the PDI values, CaT1S is the most stable, and CaTS is the least stable NANOBIN (*i.e*., CaT1S ˃CaTS1 ˃CaTS2 ˃CaT2S ˃CaTS, Fig. **S3**). Consequently, based on their PDI values (˂ 0.3), the amount of T80 present in CaT1S, followed by the amount of SDS in CaTS1 and CaTS2, may be considered optimal for long-term stability. Nevertheless, for nano-lipid formulations, a PDI value of 0.3 and below designates homogenous formulations and is regarded as satisfactory [34].

The effect of storage on ZP of NANOBINs at room temperature (25°C) was assessed for 3 months and is shown in Table **6**. The ZP values of all NANOBINs were found to decrease after 2 months of storage, except for CaTS1. A significant decrease in ZP was also observed for CaTS2 (p ˂ .05) after 2 months of storage. However, after 3 months of storage, the ZP values were found to increase in CaTS (*p ˃* .05), CaT1S (*p ˃* .05), and CaTS2 (p ˂ .05) except for CaT2S (*p >* .05) and CaTS1 (*p ˃* .05) when compared to 2 months of storage. When compared with the results of storage for 1 and 3 months, respectively, an increase in the ZP value was observed for CaTS (*p ˃* .05). The ZP value decreased slightly for CaT1S (*p ˃* .05), CaT2S (*p ˃* .05) and CaTS2 (*p =*.056). However, CaTS1 exhibited the same constant ZP value throughout the storage period. Thus, the storage period did not yield a statistically significant effect on the ZP of all NANOBINs except for CaTS2.

The effect of storage on the EE of NANOBINs was perceived and shown in Table **7**. The EE of NANOBINs was affected by the storage conditions. All NANOBINs exhibited a decrease in EE (%) during the storage period of three months at 25°C. The decrease in EE (%) may be attributed to the leakage of CAP from the particles of NANOBINs. The highest leakage of CAP was observed in CaTS, followed by CaT1S, CaT2S, CaTS2, and CaTS1 (*i.e*., CaTS>CaT1S>CaT2S>CaTS2>CaTS1). Accordingly, among all NANOBINs, CaTS1 was found to exhibit the maximum stability (minimum leakage), while CaTS exhibited the minimum (maximum leakage). Thus, the composition of CaTS1 favors the minimum leakage of CAP and maximum stability among all NANOBINs. The reduction in EE was found not to be statistically significant (*p ˃* .05) when compared to the EE of newly prepared NANOBINs.

### 3.7 Cytotoxicity Assay

NANOBINs were assayed *in vitro*, exhausting MCF-7 cells to determine their various ranges of cell death-inducing capacity using the MTT assay. The results of the percentage of cellular viability are presented in Fig. (**5**). All five formulations revealed significant cytotoxicity in the selected cells as compared to CAP alone. The results showed that CaTS1 had the highest activity, with an IC_50_ of 9.5 µg/mL, followed by CaTS2 (IC_50_ = 14.6 µg/mL), CaT1S (IC_50_ = 28.3 µg/mL), CaTS (IC_50_ = 37.4 µg/mL) and CaT2S (IC_50_ = 40.1 µg/mL) significantly (*p <* .05, *i.e*., CaTS1 ˃ CaTS2 ˃ CaT1S > CaTS ˃ CaT2S, Fig. **S4**).

## 4 DISCUSSION

The preparation of NANOBINs was carried out using the hot homogenization process. The composition of these NANOBINs consisted of more than one component, namely CAP, stearic acid (fat), sesame oil (oil), and surfactants such as T80 and SDS. The use of both non-ionic (T80) and anionic (SDS) surfactants was aimed at obtaining more stable systems [35]. The size reduction causes the formation of new surfaces for NLCs, which in turn leads to an increase in attractive forces between the particles. The increased attractive forces may lead to physical instability in the system. The surfactants, when added to the systems, may provide stability to the system by imparting repulsive forces between the particles of NLCs [36].

It was determined that surfactant type and amount had a substantial impact on the particle size of all NANOBINs. The size of NANOBINs was found to decrease when the T80 amount was increased in CaTS, CaT1S, and CaT2S (*i.e*., CaTS˃CaT1S˃ CaT2S). A noticeable effect of T80 was observed on the size reduction of NANOBINs. Makeen *et al*. [3] also observed a similar phenomenon, indicating that the incorporation of T80 led to a reduction in the size of NLCs. This finding aligns with their earlier study [9]. Nevertheless, the impact of SDS on the reduction of size, which was seen to be somewhat less efficient than that of T80. The larger-sized NANOBINs were obtained while a similar amount of SDS was used in the preparation of CaTS1 and CaTS2 as compared with CaT1S and CaT2S (*i.e*., CaTS1˃ CaT1S and CaTS2 ˃CaT2S). When the SDS amount was raised from 50 mg (CaTS) to 75 mg (CaTS1), a corresponding growth in size was seen, followed by a decrease in size upon further increase to 100 mg in CaTS2 (*i.e*., CaTS˂CaTS2˂CaTS1). The decrease in size seen with increased surfactant concentration can be due to the decrease in interfacial tension and the diffusion of surfactant molecules into the aqueous phase. These factors enable the formation of smaller-sized NANOBINs. The anionic surfactant (*e.g*., SDS) facilitates size reduction by electrostatic repulsion [37], while the nonionic surfactant (*e.g*., T80) assists in size reduction by the steric effect [38]. The combination of both surfactants (ionic and nonionic) was anticipated to have the advantages of both steric and electrostatic stabilization for obtaining efficient particle sizes during homogenization [39]. However, the smaller size of CaTS (a comparable amount of SDS and T80) than CaTS1 (more SDS than T80) and the larger size of CaT1S (more T80 than SDS) implies that T80 has a greater emulsifying capacity than SDS. The passive targeting of therapeutic substances to cancer cells was significantly influenced by particle size. The Reticuloendothelial System (RES) and the increased permeability and retention effect (EPR) were both involved in the passive targeting of nanoparticles [40]. A diameter range of 10 to 100 nm is often regarded as advantageous for cancer therapy because nanoparticles may effectively transport drugs and have increased permeability and retention (EPR) effects [41]. Two processes, phagocytosis and pinocytosis, were used to internalize the majority of nanoparticles into the cells. The particles greater than 500 nm in diameter used the phagocytosis pathway. Using the pinocytosis route, nanoparticles larger than 1 μm (macropinocytosis), between 20 and 100 nm (caveolae-mediated endocytosis), and between 120 and 150 nm (clathrin-mediated endocytosis) were internalized [42].

The PDI of nanoformulations was estimated to ensure particle aggregation or agglomeration. Furthermore, PDI provides information about the similarity of particles in the colloidal system in terms of size. A polydispersed system may be described by a PDI value of 0.5 or more (˃0.5). It may contain either particles varying more in size (*i.e*., dissimilarity in size of particles present in the finished formulations) particles in aggregated or agglomerated form in the system, or both. A polydispersed system lacks the capability to be stable for long durations of time and is hence considered unsuitable for nanotherapeutic systems. In contrast, monodispersed systems contain particles of similar sizes. A monodispersed system may be considered free from all aggregations or agglomerations. The PDI value of a monodispersed system is predicted to be closer to zero. Thus, the stability of the system, including NANOBINs, is favored by a lower value of PDI (the monodispersed system). The PDI of all NANOBINs was found to be less than 0.5, indicating their suitability and stability for longer durations of time, potentially impacting their anticancer efficacy. In contrast to small particles, aggregates do not interact with cells. The targeting effectiveness of nanoparticles in cells and tissues is reduced by aggregation or agglomeration. Additionally, the presence of undesired aggregates may result in a reduction of cellular absorption and cytotoxicity, as aggregation increases particle size while decreasing their surface area. It's plausible that these undesirable aggregates could settle out of suspension, resulting in a reduction in their bioavailability [43, 44]. The PDI and particle size are influential physical factors for endocytosis-dependent cellular uptake [34].

The Zeta Potential (ZP) refers to the electric potential difference that exists between the electric bilayer encompassing the particles, which consists of the stern and diffuse layers. The magnitude of ZP indicates the colloidal stability. A significant magnitude of ZP, whether positive or negative, generates a sufficient repulsive force that counteracts the inclination of particles to aggregate. Nevertheless, in the presence of a diminished value, the absence of a repulsive force will fail to impede the convergence and aggregation of particles, resulting in flocculation. A colloidal system with a ZP value |˃20| is considered to be electrostatically stable (no aggregation, flocculation, or phase separation, 3). Thus, the ZP of NANOBINs may be explained by the potential difference between the NLC surface and its surrounding liquid media (*i.e*., the interface between particles and liquid). All NANOBINs had a high ZP value (˃ 20), indicating electrostatically stable formulations. Among all NANOBINs, CaT1S is considered to be the most stable, while CaTS is the least stable formulation. Thus, the order of stable formulations based on the ZP value is CaT1S˃CaTS2˃CaTS1˃CaT2S˃CaTS. The ZP value of all NANOBINs was measured to be negative. The negative value of ZP is explained by the SDS and fatty acid employed in the preparation of NANOBINs that are slightly ionized. The presence of the hydroxyl ion (OH-) and hydrated oxonium ion (H3O+) leads to differential adsorption, resulting in the nonionic stabilizer T80 displaying a negative charge at the interface [44]. The surface charge of nanosystems has a significant impact on their biocompatibility, which can also change how they behave in biological systems. A few examples of the processes that the charge of the nanosystem can control include internalization, cellular membrane interactions, biological fluid stability, and opsonization. Nanoparticles with a positive charge have a higher rate of uptake compared to those with a neutral or negative charge. In contrast, it is more probable for tumor tissues to gather nanoparticles that possess a surface with a little negative charge. Contrary to neutral or negative nanoparticles, positive ones can hasten the immune system's response time [45].

DSC is a domineering method for investigating thermal behavior, including glass transition, crystallization, and melting, to assess the physical state of macromolecular materials. The physical characteristics of the lipid nanocarriers, such as crystallinity and amorphous state significantly influence the physicochemical properties of Nanostructured Lipid Carriers (NLCs) [31]. The crystallization index is a measure that quantifies the degree of organization of lipid molecules in a crystalline form inside the NLC structure. The crystallization index is important because of its influence on the physical characteristics and stability of NLC. The degree of crystallinity influences many properties of NLC, including EE [46], drug release kinetics [47], particle size, and long-term stability [48]. The decreased crystallinity may lead to faster release rates as a consequence of greater porosity and enhanced drug diffusion, as well as higher drug entrapment capacity owing to the presence of available free volume. Nevertheless, the reduced crystallinity might result in increased particle sizes as a result of the greater occurrence of disordered domains. Furthermore, the higher crystallinity of NLCs contributes to their long-term stability by preventing phase separation, aggregation, and drug expulsion. These characteristics ensure the longer shelf life of NLC. Optimizing the crystallization index of NLCs plays an important role in developing formulations that have satisfactory drug loading and release properties, particle size distribution, and long-term stability. In drug delivery applications, it enables improved control over the efficacy and performance of these lipid-based nanoparticles. The noticeable effect of surfactants was seen in the release pattern of NANOBINs. The type and amount of surfactants both visibly affected the release behavior of NANOBINs. Among all NANOBINs, CaTS was found to exhibit the lowest amount of released CAP, and this can be attributed to the presence of the least amount of surfactants (*i.e*., SDS and T80). The maximum amount of released CAP was estimated to be in CaTS1, followed by CaT1S, CaTS2, and CaT2S (*i.e*., CaTS1˃CaT1S˃CaTS2˃CaT2S˃CaTS). Thus, among all NANOBINs, the effect of surfactants on the release of CAP was observed to be maximum in the case of CaTS1. Moreover, CaTS1 exhibited better performance than CaTS2, indicating that the amount of SDS in CaTS1 is optimal for CAP release. The higher amount of SDS in CaTS2 as compared with CaTS1 is disparaging for the release of CAP from the NANOBINs. The release behavior of CaT1S was found to be better in comparison with CaT2S. The better release behavior of CaT1S can be attributed to the amount of T80 used in its preparation, which is optimal for the release of CAP. Thus, the lower amount of T80 in CaT1S than in CaT2S seems to be favorable for a better release of CAP. Consequently, the effect of SDS is more projective than that of T80 in the release of CAP from NANOBINs (SDS˃T80). In correlation with the DSC studies, CaTS1 exhibited the lowest enthalpy value (9.956 J/g) among all NANOBINs. It correlates with the highest released percentage of CAP (83%). It may be attributed to the fact that less energy was needed to break the bonds between the molecules. Moreover, CaTS (49.96 J/g) revealed the highest enthalpy value, which agrees with the lowest released value (36%). As a result, more energy was required to break the bonds, resulting in a lower release value. Given this, the percentage release of CaT1S, CaT2S, CaTS1, and CaTS2 was found to be significant (*p <* .05) when compared with CaTS.

The impact of storage conditions on the dimensions of NANOBINs was examined. The combination of two stabilizers gives more thermodynamically stable formulations. Moreover, among the stabilizers, a combination of nonionic stabilizers (*e.g*., T80) and anionic stabilizers (*e.g*., SDS) was suggested to be a better choice for achieving the desired stability [49]. Furthermore, the combination tightens the packing of stabilizer molecules because of the reduction of self-repulsion between ionic surfactant molecules [50]. The size of the obtained particles, however, continued to be in the colloidal nanometer range (< 550 nm) after three months [9]. The lack of particle clusters was demonstrated by the size increase being less than two fold. This sort of growth might be explained by the surface swelling or adsorption of additional surfactants on NANOBINs [3]. As a result, this growth corresponds to the amount of T80 used in the preparation of NANOBINs. The negative surface charge of the nanoparticles and the steric hindrance of the non-ionic surfactant can increase their stability to such a high degree [51-53].

On the other hand, CaTS1 and CaTS2 showed a contrary effect on size, with a decrease in particle size after two months of storage. However, after three months of storage, we observed an increase in particle size. Thus, after two months of storage, we found that SDS effectively reduced the particle size of NANOBINs. The decrease in size was perceived to be greater in CaTS1 in comparison with CaTS2. Therefore, the higher the amount of SDS, the more stability it provides for the particle size of NANOBINs, ensuring minimal variation in particle size during the storage period (*i.e*., CaTS2 > CaTS1). This is because the surfactants completely cover the nanoparticles. We measured the larger NANOBINs prior to the storage time, as the surfactants had not fully covered their surfaces.

During storage time, the surfactants achieved full coverage, providing ample time for effective action. Similar results were observed by Makeen *et al*., 2020 [9]. Furthermore, the size increase (*p >*.05) after three months of storage could be attributed to swelling or the adsorption of extra surfactants onto the surfaces of the NANOBINs.

The PDI values of nano-formulations may serve as an indicator of their physical stability. The long-term stability of nano-formulations favors a smaller value of PDI [3]. Nanoparticulate drug delivery systems must be stable, effective, and safe. Hence, it is critical to understand and regulate the parameters that impact the PDI of NLCs during storage [54, 55]. The storage period influenced particle size and PDI, which may cause variations in the ZP values of NANOBINs due to charge variation and particle dispersion forces. Furthermore, the tendency to agglomerate may be considered another reason for the ZP change. The ZP may be affected by numerous factors, including miscellany in particle size, amount, and type of surfactants, along with the agglomeration nature of the particles [56]. However, free esters released from lipids and surfactants might help maintain stability through steric interaction, hydration of the surface layer, and resistance to coalescence and flocculation [45]. Thus, the differences observed in particle size, Polydispersity Index (PDI), and Zeta Potential (ZP) during storage can be attributed to several mechanisms, including Ostwald ripening, agglomeration, chemical degradation, environmental factors, and physical instability [57-59].

The cytotoxicity tests are used to evaluate possible threats and ensure the safety of the developed formulations [60]. A cytotoxicity test, commonly referred to as a tissue culture assay, is used to evaluate and describe the potentially harmful effects of drugs on cells [61]. It assesses the capacity of an anticancer drug to kill cells. The cytotoxicity effects of NANOBINs and CAP alone were assayed on MCF-7 cells. Earlier studies have also demonstrated that CAP alone is incapable of inducing cell death [62, 63]. Nevertheless, NANOBINs containing both T80 and SDS enhanced the anticancer activity of CAP. The highest activity shown by CaTS1 may be attributed to the optimal quantity of T80 and SDS among all NANOBINs. Surfactants (*e.g*., T80, SDS) might potentially disrupt cellular processes by modifying the fluidity of cell membranes and interacting with them [64]. Moreover, the highest activity of CaTS1 corresponds with the release behavior. Furthermore, the significant activity exhibited by all NANOBINs could be due to tween’s capacity to block P-glycoprotein-mediated drug exocytosis [65], particle size (nano-size), and lipid nature. One study found that stearic acid and other long-chain fatty acids could interact with cell membranes to increase penetration [66]. The cytotoxicity study of CAP-loaded SLN exhibited more inhibition (95%) when compared to the suspension (72%, 22). The polymeric nanoparticles containing CAP (6.66-96.7%) were found to exhibit higher cytotoxicity than CAP alone (2.97-82.23%) in all concentrations (50-500 mg/ml), [67]. Similarly, CAP alone exhibited lower cytotoxic action when compared to CAP-loaded liposomes [68]. The half maximum concentration (IC_50_) of CAP-loaded nanoliposomes [62] and CAP-loaded PLGA-based nanoparticles [69] were found to be therapeutically more significant when compared with CAP solution. Several factors can influence the cytotoxicity study of nanoparticles containing anticancer drugs. Properties such as particle size, surface charge, composition, and drug encapsulation efficiency can significantly affect the cytotoxicity of NLCs. Smaller nanoparticles may exhibit enhanced cellular uptake and penetration, leading to increased cytotoxicity. Surface charge can influence interactions with cell membranes, affecting uptake mechanisms and intracellular drug release [70, 71]. The release profile of the anticancer drug from NLCs can impact its cytotoxicity. Controlled and sustained drug release may result in prolonged exposure of cancer cells to the drug, potentially enhancing cytotoxic effects. On the other hand, rapid drug release could lead to a burst effect, influencing the overall cytotoxicity profile [72]. The stability of NLCs in biological environments can impact their cytotoxicity by affecting drug release kinetics and cellular interactions. Factors such as storage conditions, pH, and temperature stability can influence the integrity of NLCs and, ultimately, their cytotoxic effects on cancer cells [73].

## CONCLUSION

The characterization parameters revealed excellent performance regarding EE, ZP, and particle size. All NANOBINs underwent highly commendable stability studies. The phenomenon of storage led to an increase in size, mostly caused by the swelling or adsorption of surfactants within NANOBINs that included T80 in quantities comparable to or surpassing SDS (namely, CaTS, CaT1S, and CaT2S). On the contrary, NANOBINs containing more SDS than T80 (CaTS1 and CaTS2) exhibited a decrease in size due to the complete covering of surfaces (after the storage period).

Furthermore, NANOBINs demonstrated the release of CAP for a longer period, implying that CAP will be available at the site of action for a longer duration, thus preventing the frequent administration of CAP. As a result, it can improve the patient’s acceptability. Moreover, the cytotoxicity assay exhibited that the lesser amount of CAP-loaded NLC (NANOBINs, 9.5 µg/mL, CaTS1) will have a therapeutic effect equivalent to the higher amount of CAP per se (100 µg/mL). The reduced dose of CAP will reduce its exposure to the body, thereby minimizing dose-related toxicity. So, NANOBINs (*e.g*., CaTS1) can be used in metronomic chemotherapy (*i.e*., treatment with low doses of anticancer drugs over a long time) for less severe side effects. With the evidence provided in the present study, we can conclude that NANOBINs can be considered an effective delivery method of choice for the treatment of different types of cancer, including breast cancer. In CaTS1 release and cytotoxicity experiments, the lowest enthalpy value favored the best performance. As a result, the DSC values can be utilized to forecast the *in vitro* performance of nanoformulations. Remarkably, among all NANOBINs, CaTS1 was found to give admirable exemplification based on the results of the studies performed, including release and cytotoxicity studies.

## Figures and Tables

**Fig. (1):**
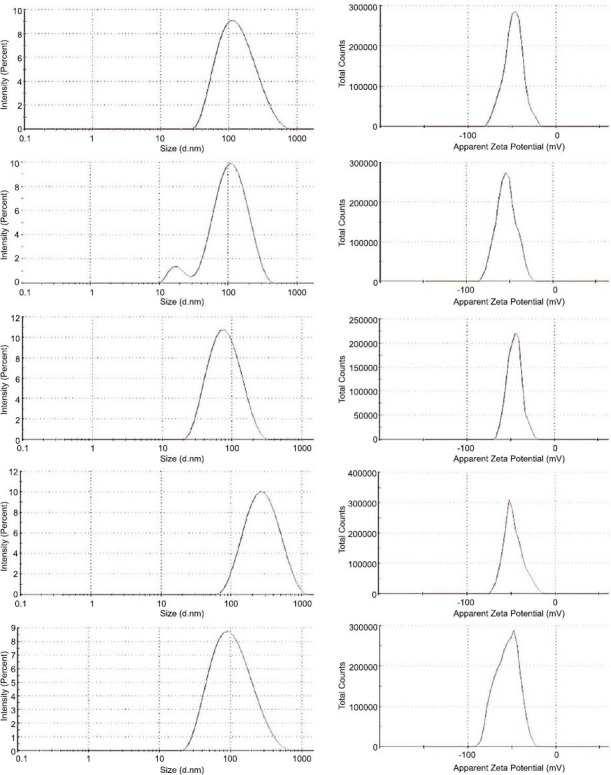
Frequency graph of size (left), and frequency graph of zeta potential (right) of NANOBINs. CaTS (first from top, size = 103.8 d.nm, Z*P =* - 48.5 mV), CaT1S (second from top, size = 85.22 d.nm, Z*P =* - 54.0 mV), CaT2S (third from top, size = 61.51 d.nm, Z*P =* - 45.8 mV), CaTS1 (fourth from top, size = 223.1 d.nm, Z*P =* - 47.6 mV), CaTS2 (fifth from top, size = 81.92 d.nm, Z*P =* - 55.5 mV).

**Fig. (2):**
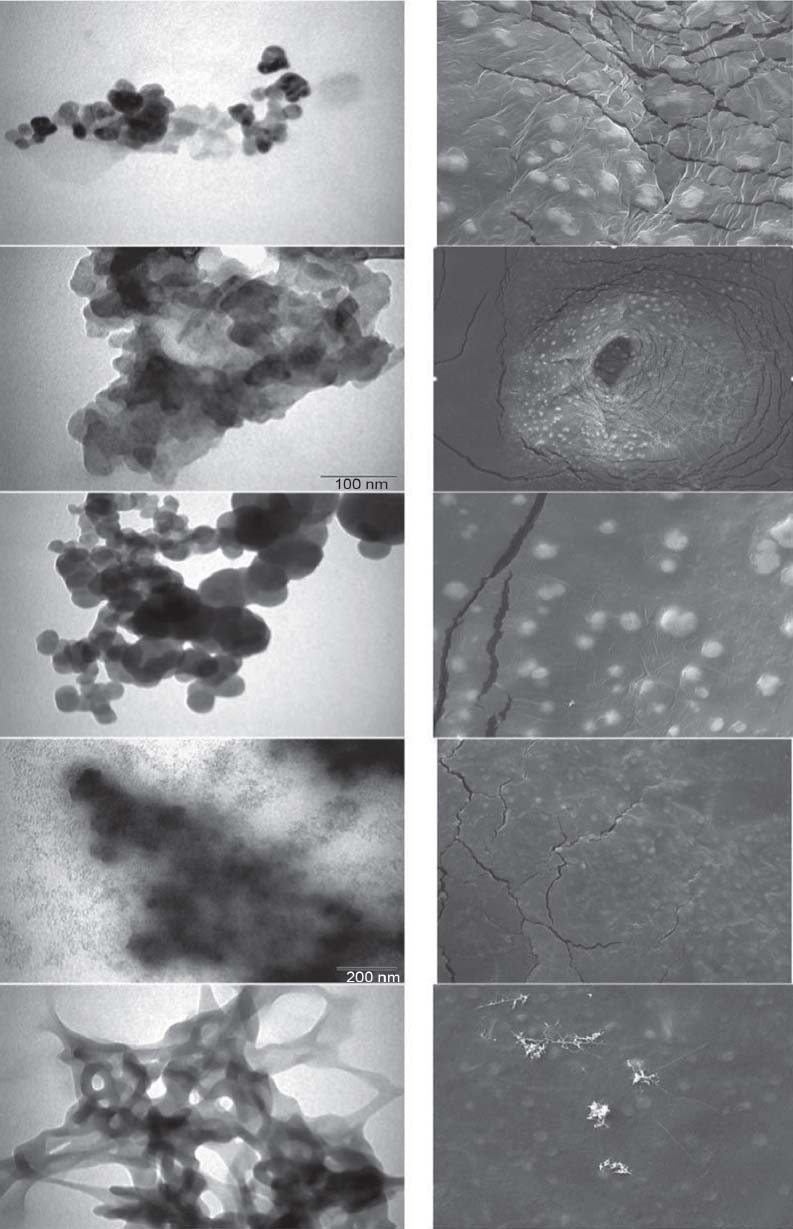
TEM (left) and SEM (right) images of NANOBINs. CaTS (first from top), CaT1S (second from top), CaT2S (third from top), CaTS1 (fourth from top), CaTS2 (fifth from top).

**Fig. (3):**
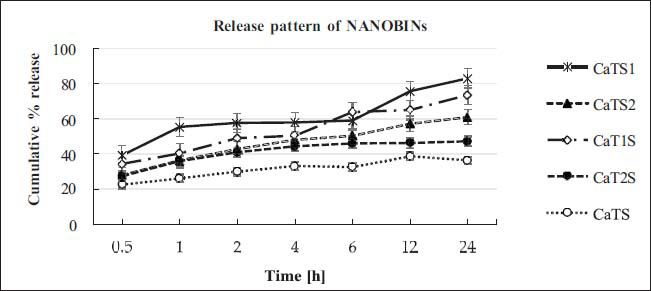
Release pattern of NANOBINs.

**Fig. (4):**
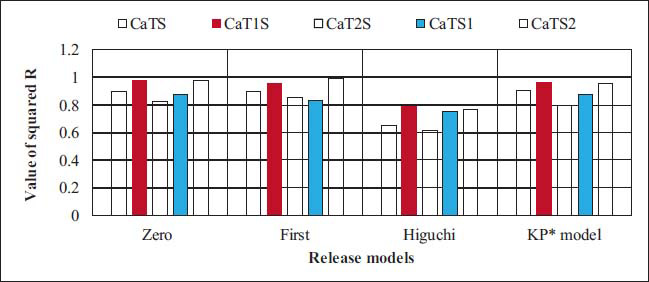
Release mechanism of NANOBINs.

**Fig. (5):**
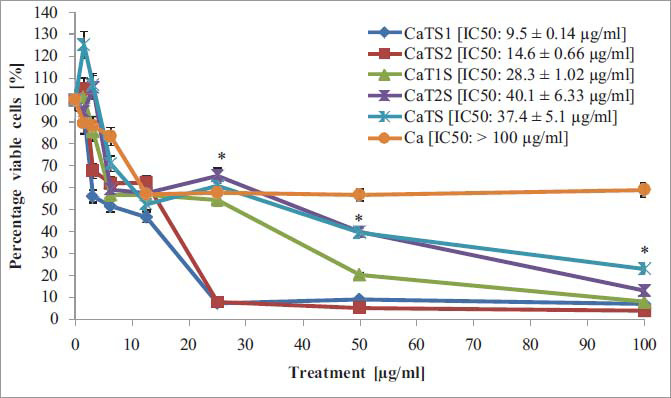
Cytotoxicity assay of CAP per se and NANOBINs. The average across three replicates is used to represent values.

**Table 1:** Amount of surfactants used in various NANOBIN formulations.

**NANOBINs**	Tween-80 (µL)*	**Sodium Dodecyl Sulphate (SDS, mg)**
CaTS	50	50
CaT1S	75	50
CaT2S	100	50
CaTS1	50	75
CaTS2	50	100

*Density = 1.07 g/cm^3^.

**Table 2:** Results of size, PDI, ZP, and EE of NANOBINs.

**NANOBINs**	**Size (d.nm)** **(*n =* 3, ±SD)**	**PDI** **(*n =* 3, ±SD)**	**ZP (-ve, mV)** **(*n =* 3, ±SD)**	**EE (%)** **(*n =* 3, ±SD)**
CaTS	98.01 ± 8.52	0.323 ± 0.09	46.47 ± 2.10	96.071 ± 0.386
CaT1S	86.64 ± 1.84	0.297 ± 0.00	61.87 ± 6.83	95.098 ± 0.770
CaT2S	65.50 ± 4.68	0.296 ± 0.04	47.47 ± 1.70	96.539 ± 0.707
CaTS1	193.27 ± 25.93	0.370 ± 0.08	51.90 ± 3.73	96.640 ± 0.916
CaTS2	120.31 ± 64.00	0.260 ± 0.03	61.47 ± 3.62	94.121 ± 0.184

**Table 3:** Thermal analysis (DSC) of different materials.

**NANOBINs**	**Onset Temperature (°C)**	**Peak Temperature (°C)**	**Enthalpy (J/g)**
CAP	121.34	122.58	35.293
Stearic acid	54.59	57.32	222.5
Mannitol	166.58	168.68	258.27
CaTS	165.66	166.77	49.96
CaT1S	165.8	166.82	31.55
CaT2S	165.47	166.37	26.43
CaTS1	165.41	166.46	9.956
CaTS2	164.82	165.93	39.404

**Table 4:** Effect of storage period on size (d.nm, *n =* 3, ±SD) of NANOBINs.

**NANOBINs**	**Month**		
	**1**	**2**	**3**
CaTS	98.01±8.52	97.01±13.96	124.07±8.70
CaT1S	86.64±1.84	92.37±4.28	119.67±12.95
CaT2S	65.50±4.68	85.90±7.002	100.87±10.095
CaTS1	193.27±25.93	108.52±18.25	119.17±32.98
CaTS2	120.31±64.00	86.83±6.53	94.15±10.86

**Table 5:** Effect of storage period on PDI (*n =* 3, ±SD) of NANOBINs.

**NANOBINs**	**Month**		
	**1**	**2**	**3**
CaTS	0.323±0.09	0.31±0.09	0.40±0.02
CaT1S	0.297±0.00	0.33±0.06	0.24±0.02
CaT2S	0.296±0.039	0.26±0.012	0.37±0.042
CaTS1	0.37±0.08	0.355±0.107	0.28±0.08
CaTS2	0.27±0.03	0.24±0.01	0.29±0.09

**Table 6:** Effect of storage period on ZP (-ve, mV, *n =* 3, ±SD) of NANOBINs.

**NANOBINs**	**Month**		
	**1**	**2**	**3**
CaTS	46.47±2.10	42.13±4.74	47.53±1.76
CaT1S	61.87±6.83	49.57±5.26	51.33±6.93
CaT2S	47.47±1.70	45.33±4.84	44.17±1.42
CaTS1	51.9±3.73	52.5±1.78	51.67±3.65
CaTS2	61.47±3.62	45.10±3.58	53.50±3.73

**Table 7:** Effect of storage period on EE (%, *n =* 3, ±SD) of NANOBINs.

**NANOBINs**	**Month**		
	**1**	**2**	**3**
CaTS	95.60±3.04	95.14±2.57	94.67±1.14
CaT1S	94.75±1.14	94.41±0.46	94.07±0.25
CaT2S	95.74±0.72	95.50±1.67	95.25±1.42
CaTS1	96.58±0.79	96.52±1.22	96.46±0.64
CaTS2	93.91±1.44	93.69±2.28	93.47±1.13

## Data Availability

The data and supportive information are available within the article.

## LIST OF ABBREVIATIONS

CAP/Ca: Capecitabine
CaTS/CaT1S/ CaT2S/CaTS1/ CaTS2: Different types of NANOBINs
CI: Crystallization Index
EE: Entrapment efficiency
NANOBIN: NLC containing capecitabine
NLC: Nanostructured lipid carriers
PDI: Polydispersity Index
SEM: Scanning Electron Microscopy
TEM: Transmission Electron Microscopy
ZP: Zeta Potentia
