# Supplementary material for: Capecitabine-loaded NLC for Breast Cancer Treatment: Preparation, Characterization, and In vitro Evaluation
Source: Curr Drug Deliv. 2024 Jul 29;22(7):968–82. doi: 10.2174/0115672018309370240708113038 (PMC12606612; doi:10.2174/0115672018309370240708113038)
Supplement: Supplementary file 1 [file CDD-22-7-968_SD1.pdf]

SUPPLEMENTARY MATERIAL

Capecitabine-loaded NLC for Breast Cancer Treatment: Preparation, Characterization, and *In vitro* Evaluation

Muhammad Hadi Sultan[1,#], Yosif Almoshari[1,#], Syam Mohan[2], Mohamed Ahmed Al-Kasim[3], Hamad S. Alyami[4], Mohammad Azam Ansari[5] and Mohammad Intakhab Alam[1,*]

[1]Department of Pharmaceutics, College of Pharmacy, Jazan University, Jazan, Saudi Arabia; [2]Substance Abuse and Toxicology Research Centre, Jazan University, Jazan, Saudi Arabia; [3]Department of Pharmacology, College of Pharmacy, Jazan University, Jazan, Saudi Arabia; [4]Department of Pharmaceutics, College of Pharmacy, Najran University, Najran, Saudi Arabia; [5]Department of Epidemic Disease Research, Institute for Research and Medical Consultations [IRMC], Imam Abdulrahman Bin Faisal University, Dammam, Saudi Arabia

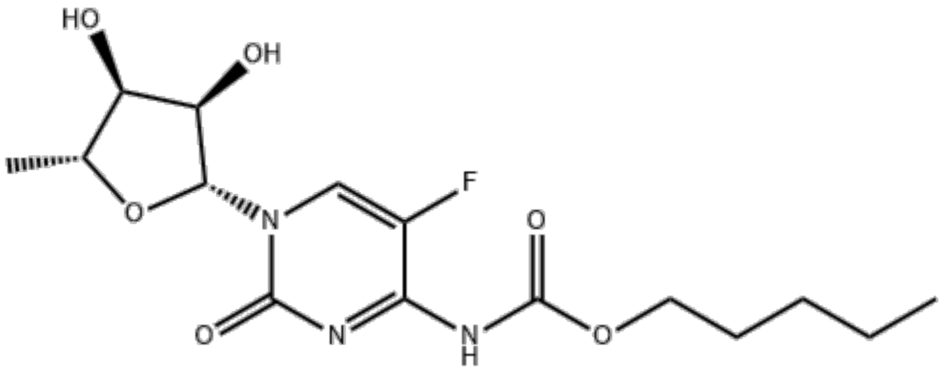

Figure S1: Chemical structure of capecitabine ($C_{15}H_{22}FN_3O_6$).

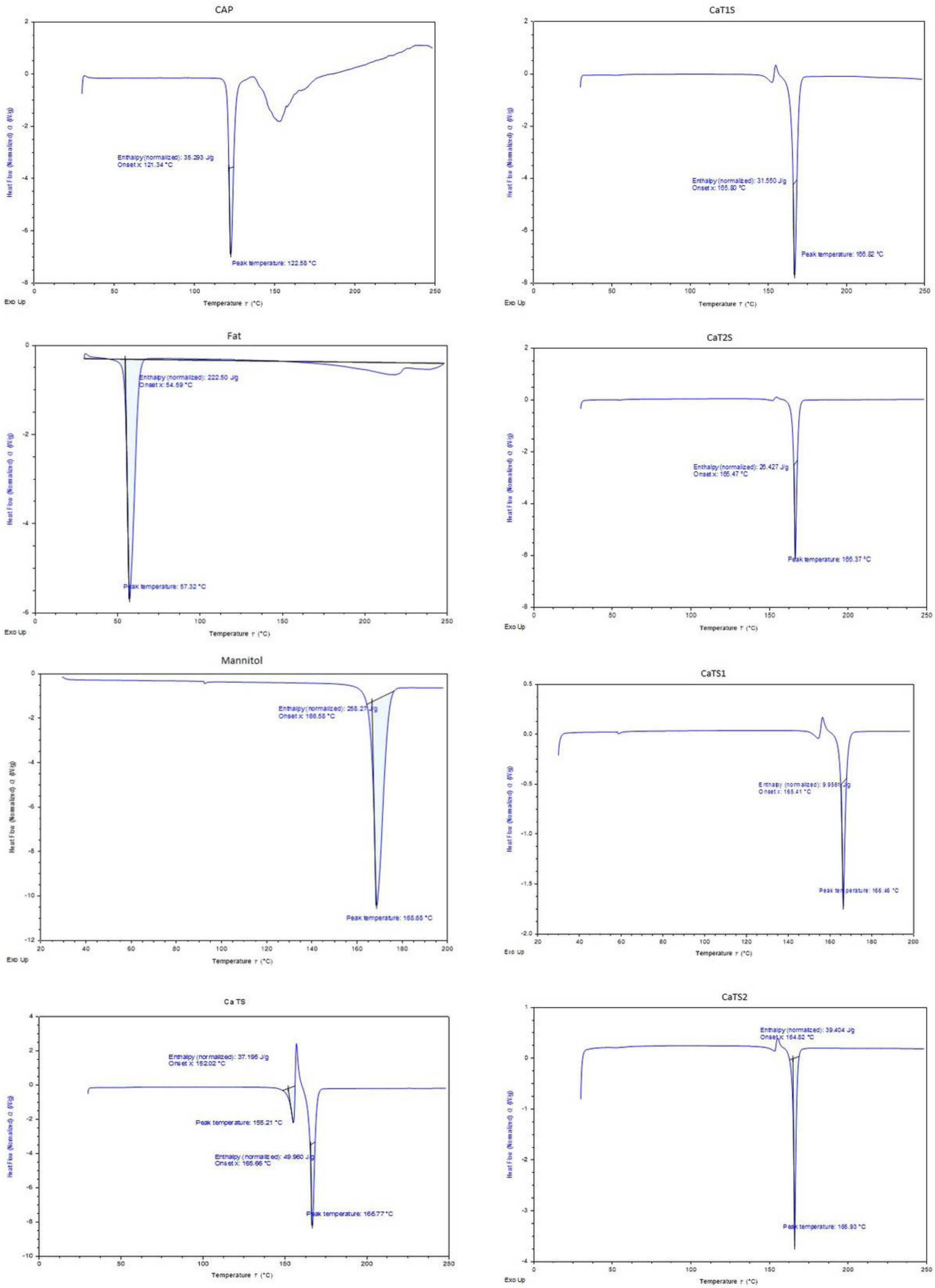

Figure S2: DSC thermograms of CAP, stearic acid (fat), mannitol, and NANOBINs (CaTS, CaT1S, CaT1S, CaTS1, CaTS2).

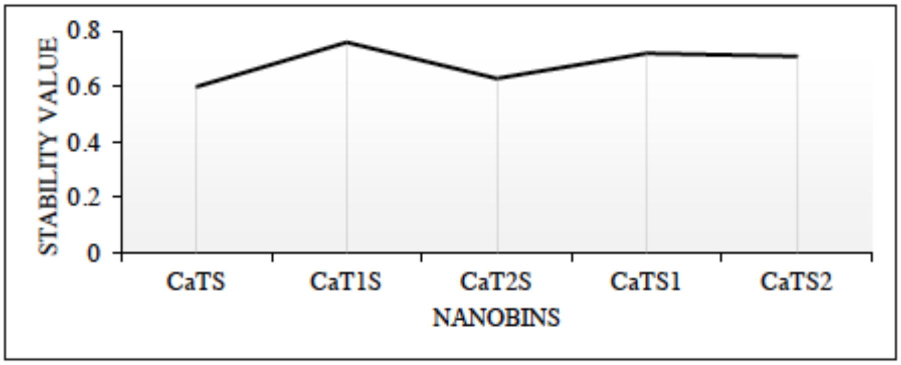

Figure S3: NANOBIN's stability based on PDI. Among all NANOBINs, CaT1S was determined to be the most stable. The stability value was determined by deducting the PDI value from unity.

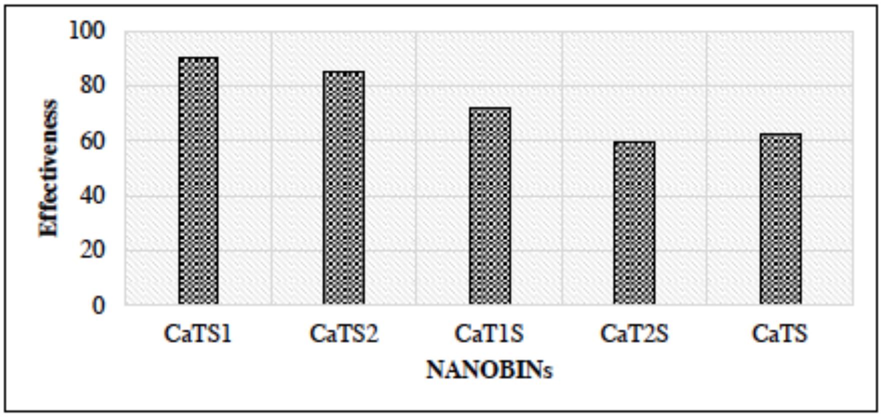

Figure S4: Efficacy of NANOBINs against cytotoxicity studies. Among all NANOBINs, CaTS1 and CaT2S were observed to be the most and least effective, respectively, for the treatment of cancer.
